# Use of a CME workshop to introduce and promote the specialty of Family Medicine in Ethiopia 

**DOI:** 10.4102/phcfm.v3i1.226

**Published:** 2011-08-05

**Authors:** Prosper M. Lutala

**Affiliations:** 1 Department of Family Medicine, University of Goma, Congo (DRC)

## To the Editor

I read with profound interest the article entitled, *‘Use of a CME workshop to introduce and promote the specialty of Family Medicine in Ethiopia’*, authored by Jane Philpott and Miliard Derbew.^1^ They used continuing medical education (CME) as an innovative way to introduce the concept of Family Medicine (FM) in a medical community in Ethiopia. After different sessions followed by discussions and assessments, the authors introduced the concept which led to the listing of roles, skills and values that could characterise a Family Physician in Ethiopia. In addition, the action steps which could lead to the launch of the specialty in Ethiopia were identified. 

Several other routes have been followed in different FM programmes throughout Africa.^2,3,4,5^ In the Congo or DRC (The Democratic Republic of the Congo), for example, after MAP (Medical Assistance Programs) International’s consultations with hospitals from Kenya and Zaire (former DRC) in 1995 resolved to start a Family Medicine postgraduate programme,^2^ further consultations were held by each country separately with each country to formalise the idea. Congo opted for joint CME through an NGO (Non-government organisation) the ‘Doctors on Call for Service’, a church (Inter-church organisation, *‘Eglise du Christ au Congo’* [The Christian Church of the Congo]), and a Southern African university (Medical University of Southern Africa) joint‘s CME. This led, in 1997, to the start of a satellite distance learning master’s course. Nine years later, the first department of Family Medicine was launched at the University of Goma. On the other hand, Kenya opted for partnerships with faith-based health institutions, universities (firstly, Moi University and recently, Aga Khan University), the Ministry of Health and civil society (the Institute of Family Medicine, INFAMED, and The Christian Health Association of Kenya, CHAK) to start Family Medicine in 2005.^2,3^ In the first case of Congo (DRC), recognition and accreditation are still on hold with a serious shortage of staff whilst the Kenyan scenario is going well in uptake, government support, accreditation and the career path of a graduated Family Physician (FP) well defined. The Rwandan^6^ case-study of 1999 is similar to the Kenyan’s in its history and is more promising in terms of the acceptance and career path of graduates despite the fact that local faculties are still scarce. The case of Uganda,^4^ especially at Makerere University, can give more insight. 

A Canadian Family Physician with a Canadian grant from the Canadian International Development Agency, started a Family Medicine programme at Makerere University in 1989. After he left in 1994 the programme collapsed because of a lack of funds, unstable leadership at the medical school and resistance from other specialists.^4^ The programme was later reactivated; however, problems are still pending. The government remains unclear on the specific roles that family physicians have to play in the health system which have to be defined and formalised in order for the discipline to be accepted and to become attractive. In all of the above-mentioned countries the recent contribution of Belgium through the project of VLIR (Vlaamse Interuniversitaire Raad) in strengthening FM in all these countries cannot be overemphasised. 

Despite the growing number of countries involved in the training of Family Physicians, few have documented their path before the real implementation. Some programmes are still struggling either for the graduates’ recognition and/or for an official accreditation of training sites. Hence, a question that arises is, ‘which of these approaches have the potential to achieve a quick accreditation?’ 

At this point, the speed in accreditation cannot be predicted; nonetheless, it will depend on several factors. These factors include the manner in which Family Medicine fits into the local health system, how it has been negotiated with all stakeholders, the way it responds to the needs raised which are partially or not at all addressed by existing disciplines, and finally how its products (graduates) are making a difference on ground level. All others factors aside, we can assume from Kenyan and Rwandese case-studies^2,3,5^ that collaboration of colleagues from complementary agencies was a key for success in the accreditation process. Medical training has to be covered by an university, preferably a local one; the Ministry of Health and Professional Bodies have to prepare a conducive environment for practicals and work, and finally civil societies and funding agencies have to play a catalytic role in funding, utilisation of products and lobbying.^2,3^ 

Secondly, let us examine contributions made by colleagues in different departments to support the development of FM Departments. In this case, the long-term roles for those ‘outsider’ specialists assisting implementation of FM training have to be defined or anticipated once adequate numbers of FP have been trained and are fulfilling their roles as health providers, researchers and teachers. If the intention is to assist in launching FM, what is the appropriate time to hand over to new graduates in FM? 

Who are the right stakeholders to involve in the early stage of training? To what extent should they be involved? Anecdotal reports in some programmes argue that strong involvement of an ‘outsider’ specialist sometimes slows down the process of departmental progress. The result is detrimental to maturation, autonomy and the recognition of FM. 

McWhitney^6^ supported the notion that to be recognized as an autonomous specialty, a field has to fulfil three requirements namely, (1) methodology, (2) epistemology and (3) practicals. Therefore, Family Medicine implemented by ‘outsider’ specialists and even defined by them, does not have the ability to nurture in its traditional philosophy. The same can be questioned about research output and Objective Structured Clinical Examination (OSCE). 

Thirdly, there is a need to explore the chances of success of networking departments in neighbouring African regions instead of relying entirely on ‘foreign’ specialists. The *success* of this networking will depend firstly, on acceptance of the discipline of FM by existing stakeholders on the ground (which could depend on their involvement). Secondly, it will depend on cross boundaries exchange, and finally on commitment and the availability of lecturers between countries. In Africa, however, where academics are underpaid and departments, especially FM, are underfunded, the payment burden for visiting lecturers can be foreseen. What can the initiators of FM departments in Africa do to overcome those constraints? Or, how feasible is the involvement of external lecturers within the financial resilient mechanisms of each institution? 

In conclusion, Africa is moving in the right direction in the FM implementation process with regard to evidence-based decisions in response to practical issues. This could be the only way to give countries a ‘home-grown’ and ‘locally owned’^7^ concept of Family Medicine. However, some common practical issues encountered in the process have to be shared and the roles of the ‘insiders’ and ‘outsiders’ should be clearly outlined throughout the process. 
